# Editorial

**Published:** 1917-02

**Authors:** 


### EDITORIAL

The Atlanta Journal-Record of Medicine has its Business Office at 23 West Alabama Street where all business communications should be sent.

Remittances are payable to The Journal-Record of Medicine.

Reprints of Original Articles will be furnished at cost price. Requests for them had best be made on the Manuscript.

Upon request, we will present, postpaid, to each contributor of an original article, twenty copies of The Journal-Record of Medicine containing such article.

### AN AMAZING PROPHECY OF WAR.

This article, quoting a war prophecy said to have been made over 300 years ago, is reprinted in the London Newspapers from the London Daily Call of October 22.

The origin of this amazing document is not very clear, but the manuscript, in Latin, was found among the papers of the late Odrien Peladen, author of a work on ''Prophecies,'' and the editor of a review, ''The Annals of the Supernatural.'' It was found by his son, who had it translated into French, and it was published in the ''Figaro.''

According to the son, M. Peladen came into possession of the manuscript through a monk of Saint-Michael de Frigolet, near Tarascon, who, in turn, received it from Abbe Donat, a learned priest, who died at a very great age.

### Allegory Deciphered.

In deciphering the allegory it must be remembered that

France is represented by a cock.
England is represented by a Leopard.
Russia is represented by a White Eagle·
Germany is represented by a Black Eagle.''
Austria is represented by the ''Other Eagle.''
The Lamb stands for Justice, Mercy and Truth.

**Here is the prophecy in full:**

1. Several times has one seemed to recognize him, because all the slayers of the Lamb resemble each other, and all the wicked are the persecutors of officers, and princes will receive burial, for to the carnage caused by firearms will be added those who perished by famine and plague.

2. The veritable Anti-Christ will be one of the monarchs of his time, a son of Luther; he will invoke God and call himself his messenger.

3. The prince of lies will swear by the Bible; He will call himself the arm of the Most High, chastising corrupted people.

4. He will have only one arm, but his innumerable armies, who will take as their motto, "God is with us," will seem like infernal legions.

5. For a long time he will act by ruse and treason; his spies will spread over all the earth; and he will be the master of the secrets of those in power.

6. He will have theologians in his pay to certify and prove his celestial mission.

7. A war will furnish him with the reason for lifting the mask. It will not be one which he will make against the French monarch, but another which will be easily recognized by the fact that in two weeks' time it will have become universal.

**Will Carry Eagle.**

8. It will call to arms all Christians, all Mohamedans, and other very distant peoples. Armies will be formed in the four parts of the world.

9. For men's minds will be opened by angels, and in the third week they will understand that this is the anti-Christ, and that they will all become slaves, if they do not trample down this conquering one.

10. The Anti-Christ will be recognized by several marks; he will chiefly massacre priests, monks, women, children and old people. He will show no mercy; he will pass along holding a torch like the barbarians, but invoking the name of Christ.

11. His false words will resemble those of the Christians, but his acts will be those of Nero and the Roman prosecutors; there will be an eagle in his coat of arms, and there will also be one in that of his confederate, the other wicked monarch.

**The Pope's Bull.**

12. But this one is a Christian, and he will die cursed by Pope Benedictus, who will be elected at the beginning of the reign of the Anti-Christ.

13. Priests and monks will noe longer be seen confessing and absolving the combatants, because for the first time priests and monks will fight with the other citizens, but also because, Pope Benedictus having cursed the Anti-Christ, it will be proclaimed that all those who wage war against him will be in a state of grace, and should they die, will, like martyrs, go straight to heaven.

14. The Pope's "Bull" proclaiming these things will make a great sensation, and will cause the death of the monarch, and the Anti-Christ's ally.

15. In order to conquer the Anti-Christ, more men must be killed than Rome has ever held. It will require an effort from all lands, for the Cock, the Leopard, and the White Eagle would not suffice to overcome the Black Eagle if they were not helped by the prayers and devotion of all the human race.

16. Never before has humanity been in such peril, for the triumph of the Anti-Christ would be that of the Demon, in whom is incarnated.

17. For it has been said that 20 centuries after the incarnation of the Word, the Beast in his turn would be incarnated, and would threaten the earth with as many evils as the Divine Incarnation brought it graces.

**Britain The Ally.**

18. Near the year Two Thousand the Anti-Christ will appear; his army will surpass in numbers anything herebefore imagined; there will be Christians among his hordes, and amongst the defenders of the Lamb there will be Mohamedans and savage tribes.

19. For the first time the Lamb will be entirely red, in the whole of the Christian world there will not be a space that will not be red; and the heavens, the earth, the water, and even the air, will be red, for blood will flow in the sphere of the four elements at the same time.

20. The Black Eagle will throw itself upon the Cock, which will lose many of its feathers, but will strike heroically with its spurs. It would be soon annihilated were it not for the help of the leopard and its claws.

21. The Black Eagle, which will come from the land of Luther, will surprise the Cock by another side, and will invade one half of the land of the Cock.

**Russian Invasion.**

22. The White Eagle, which will come from the land of the north, will surprise the Black Eagle, and will completely invade the land of the Anti-Christ from one end to the other.

23. The Black Eagle will be forced to leave the Cock to fight the White Eagle, and the Cock will pursue the Black Eagle into the land of the Anti-Christ to help the White Eagle.

24. The battles waged until then will be small in comparison to those that will take place in the land of Luther, because the seven angels will at the same time pour fire from their burners on the impious land (image taken from the Apocalypse), which means that the Lamb will order the extermination of the Anti-Christ's race.

25. When the Beast sees he is lost he will become furious; during months the beak of the White Eagle, the claws of the Leopard and the spurs of the Cock must harass him.

26. Rivers will be crossed over masses of dead bodies, which in some places will change the course of the waters. Only great noblemen, superior the Great Wicked One.

27. The Anti-Christ will several times ask for peace, but the seven angels who precede the three animals, defenders of the Lamb, have declared that victory shall only be accorded on the condition that the Anti-Christ be crushed, like straw on the threshing-floor.

28. Executors of the Lamb's justice, these three animals cannot stop fighting as long as any soldiers remain to the Anti-Christ.

29. The reason the sentence of the Lamb is so implicable as that the Anti-Christ has pretended to be a Christian and to be acting in His Name, so that if he did not perish, the fruit of the Redemption would be lost, and the gates of Hell would prevail against the Savior.

**End of the Anti-Christ.**

30. It will be seen that it is not a human combat which will be waged where the Anti-Christ forges his arms. The three animals, defenders of the Lamb, will exterminate the Anti-Christ's last army; but the battlefield will become an altar of sacrifice, larger than the greatest of cities, and the corpses will have changed its shape by raising in it chains of mounds.

31. The Anti-Christ will lose his crown, and will die demented and alone· His empire will be divided into 21 states, but none will have either a royal house, an army, or vessels.

32. The White Eagle, by Michael's order, will drive the Crescent from Europe, where only Christians will remain; it will occupy Constantinople.

33. Then an era of peace and prosperity will commence for all the universe, and there will be no more war, each nation being governed according to its wish and living in justice.

34· There will be no more Lutherans or Schisnatics. The Lamb will reign, and the joys of humanity will commence. Happy they who, escaping from the perils of the prodigious time, can taste of the fruit, which will be the reign of the Eternal Spirit, and the sanctification of humanity, only to be achieved by the defeat of the Anti-Christ.—The Above was Taken from the Kalamazoo Gazette, Thursday, December 22, 1914.

